# Temperature-controlled, chromatography-free synthesis of allobetulin derivatives via trifluoroacetic acid-mediated Wagner-Meerwein rearrangement

**DOI:** 10.1016/j.mex.2026.104115

**Published:** 2026-08-25

**Authors:** Yanli Wang, Panpan Sun, Li Wang, Xiwei Zhao, Gang Huang, Xiaofei Hao, Peng Liu, Qiang Wang

**Affiliations:** a School of Chemical Engineering, Zhengzhou University, Zhengzhou, 450001, China; b Institute of Reproductive Health, Henan Academy of Innovations in Medical Science, Zhengzhou, 450000, China; c School of Medicine, Huanghe Science and Technology College, Zhengzhou, 450063, China; d Institute of Chemistry, Henan Academy of Sciences, Zhengzhou, 450002, China; e Quality Inspection and Analysis Testing Research Center, Henan Academy of Sciences, Zhengzhou, 450002, China

**Keywords:** Allobetulin derivatives, Wagner-Meerwein rearrangement, Trifluoroacetic acid, Temperature-controlled selectivity, Chromatography-free synthesis

## Abstract

Allobetulin derivatives are bioactive lupane-type triterpenoids with promising antiviral, anticancer and anti-inflammatory activities. Conventional synthetic routes rely on harsh acidic conditions and tedious column chromatography purification, leading to low scalability and significant product loss. This study describes a streamlined, one-pot protocol for the synthesis of five allobetulin derivatives from readily available betulin, betulinic acid or betulonic acid, using trifluoroacetic acid (TFA) as a dual-function catalyst and reaction medium. Product selectivity is precisely controlled by reaction temperature, eliminating the need for modifying the reagent system. The method delivers 90–95% isolated yield without chromatographic purification, and the crude product purity exceeds 95% by NMR analysis.

• Temperature-dependent selectivity enables access to either C3 free alcohols or C3 trifluoroacetate esters without altering the reaction formulation
• Chromatography-free workup minimizes product loss and simplifies isolation for preparative-scale synthesis
• Broad substrate scope covering three representative lupane-type triterpenoid scaffolds

## Graphical abstract

**Figure:**
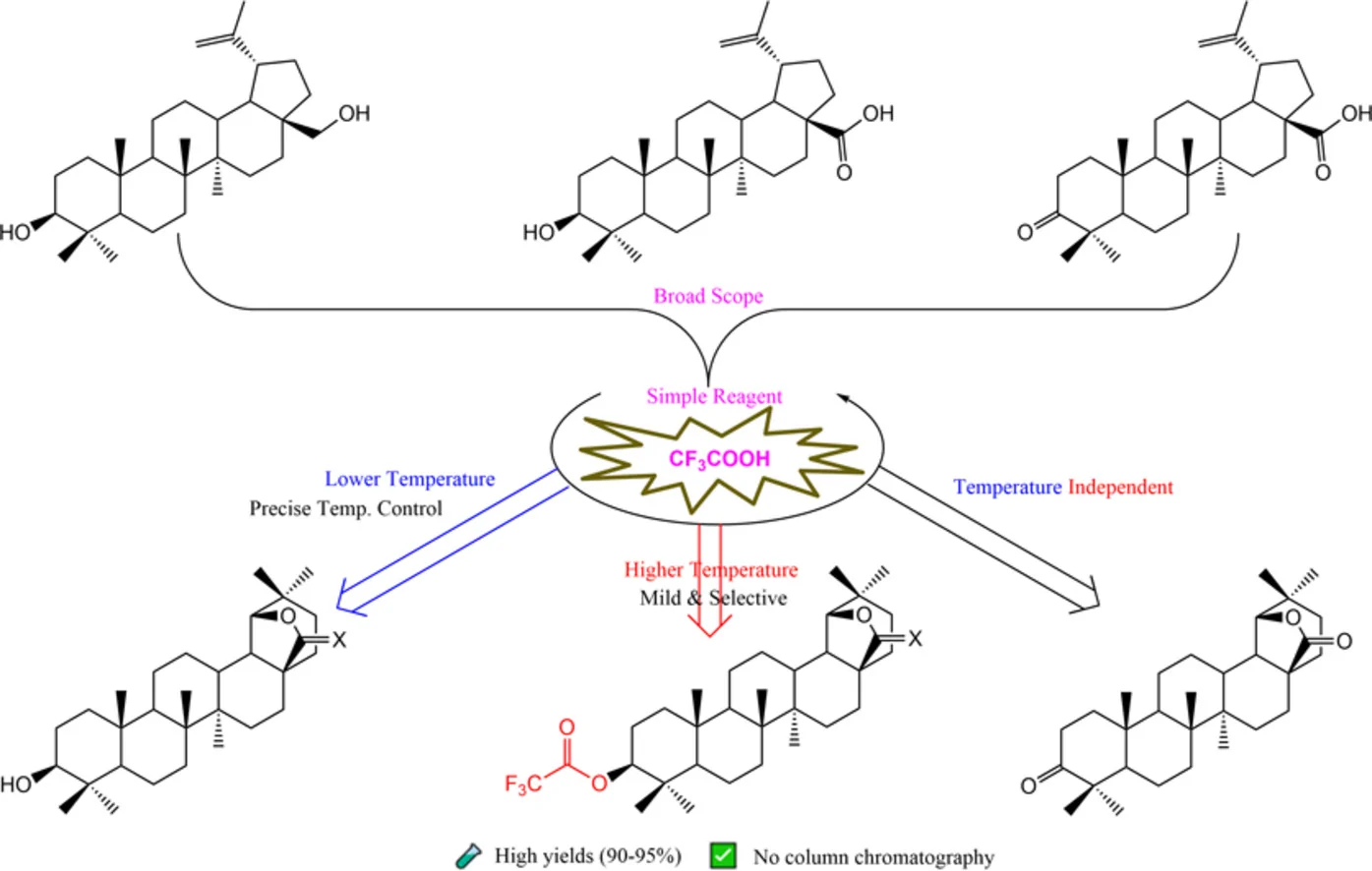

**Specifications table**

**Table:** 

**Subject area**	Chemistry
**More specific subject area**	Organic Synthesis; Natural Product Derivatization
**Name of your method**	Wagner-Meerwein Rearrangement
**Name and reference of original method**	L. Borkova, S. Gurska, P. Dzubak, R. Burianova, M. Hajduch, J. Sarek, I. Popa, M. Urban, Lupane and 18α-oleanane derivatives substituted in the position 2, their cytotoxicity and influence on cancer cells, Eur. J. Med. Chem. 121(2016)120–131. https://dx.doi.org/10.1016/j.ejmech.2016.05.029
**Resource availability**	All reagents are commercially available from standard suppliers. Raw NMR data and detailed spectral assignments are available from the corresponding author upon reasonable request.

## Background

Allobetulin, the 18α-oleanane (germanicane-type) structural isomer of betulin generated via Wagner-Meerwein rearrangement, is a privileged scaffold for the development of bioactive oleane-type triterpenoid derivatives [1]. Prior studies have demonstrated its potent antiviral, anticancer and anti-inflammatory activities [2], and further reports show that C3-modified allobetulin derivatives - including 3-O-trifluoroacetate esters – can exhibit improved profiles such as antioxidant activity [3]. Beyond its documented bioactivities, allobetulin serves as a crucial synthetic intermediate and a valuable conformational probe. Its distinct 18α-H configuration, in contrast to the naturally prevalent 18β-H (oleanane) series, imparts unique stereoelectronic properties that are of interest for structural diversification and mechanistic studies. These attributes collectively underscore the need for efficient, scalable routes to allobetulin and its C3-functionalized analogues..

Traditional synthetic routes to allobetulin predominantly rely on Brønsted acids [[4], [5], [6]] (e.g., 4-methylbenzenesulfonic acid, formic acid, Amberlyst 15 as a solid-phase sulfonic acid) or solid Lewis/ Brønsted acids [7] (eg., Montmorillonit-K10, which presents both Brønsted and Lewis acids), or Lewis acids [1,8] (eg., bismuth (III) salts, and ferric trichloride, etc.). These conditions often entail higher temperatures, prolonged reaction times, low yield, and tedious work-up procedures, with product isolation still depending on column chromatography – leading to material loss that penalizes scale-up.

Initial experiments in our laboratory aimed at the selective protection of C-3 hydroxyl in betulin while preserving the C-28 hydroxyl. Unexpectedly, when TFA was employed to remove the 4,4′-dimethoxytrityl (DMTr) protecting group, it not only effected rapid deprotection but also promoted a concomitant Wagner-Meerwein rearrangement, delivering the corresponding allobetulin derivative in remarkably high yield with no observable side products. This serendipitous observation-documented in our concurrent communication [9] – prompted us to evaluate TFA as a potential acid catalyst for the efficient synthesis of allobetulin. Building on this insight, we systematically examined the generality of TFA-mediated rearrangement across a range of lupane-type triterpenoids, including betulin, betulinic acid, and betulonic acid. Among these, betulonic acid is of particular interest: a lupane-type pentacyclic triterpenoid found in diverse plant species (e.g., *Liquidambar styraciflua, Eucalyptus viminalis*) and readily accessible via semisynthesis from betulin [10]. Recent comprehensive reviews further highlight its broad spectrum of biological activities-including anti-tumor, anti-viral, anti-inflammatory, and anti-bacterial effects-and its emerging potential as a privileged scaffold for triterpenoid-based therapeutics [11].

Here we exploit trifluoroacetic acid (TFA) itself as the sole reagent. TFA acts as a precise temperature controlled Brønsted acid to promote the rearrangement, while temperature alone switches the outcome between the C3-free alcohol (lower temperature) and the C3-O-trifluoroacetate ester (ambient temperature). The protocol is one-pot and chromatography-free, delivering 90–95% isolated yield. By doing so, it directly supplies either the allobetulin scaffold or its C3-activated trifluoroacetate intermediate (ready for downstream substitution/transesterification or biological evaluation) in a single operational step from betulin, addressing the above practical bottlenecks.

## Method details

### General materials and instrumentation

All reagents for synthesis were purchased commercially and used without further purification. The progress of reactions was monitored by thin-layer chromatography (TLC) on silica gel plates, visualized under idione vapor. Typical eluent was ethyl acetate/petroleum ether (EA/PE = 1:7). Chloroform-*d* (CDCl_3_, 99.9% deuterium, CIL) was purchased from Qingdao Tenglong Weibo Technology Co. Ltd. (Qingdao, China). ^1^H and ^13^C NMR spectra were recorded on a JNM-ECZ 400 s and an Agilent 400 MR NMR spectrometer. Chemical shifts are reported in parts per million (*δ* ppm) relative to the residual solvent peak (*δ* 77.0 for ¹³C) or TMS (*δ* 0.00 for ¹H), and coupling constants (*J*) are given in hertz (Hz). High resolution mass spectrometry (HRMS) was excuted utilizing a ThermoFisher Orbitrap Exploris 120 mass spectrometer and a Shimadzu LCMS-9050 Q-TOF mass spectrometer.

### Optimization of reaction conditions

The reaction conditions, including solvent, TFA amount, and temperature, were systematically evaluated to balance reactivity, cost, operational simplicity, and scalability. For solvent screening, dichloromethane (DCM) proved optimal: alcohols are incompatible with TFA due to side reactions; DMSO and DMF incur high costs and severe work-up burdens; ethers (THF, dioxane) and esters (EtOAc) risk acid-catalyzed side reactions; acetone and acetonitrile are economically unfavorable for scale-up. DCM uniquely combines low cost, a low boiling point (39.8 °C), and facile work-up—simple washing with water and saturated NaHCO₃ followed by evaporation directly affords the product, with no need for column chromatography. For TFA stoichiometry, using 0.2 mL (ca. 2.6 equiv) per 1 mmol of substrate achieved full conversion across all tested lupane-type triterpenoids. While increasing TFA to 10 equiv or higher drastically accelerated the reaction (C3-OTFA ester formation within 5 min at room temperature), it introduced unnecessary reagent waste and increased work-up complexity; thus, 2.6 equiv represents the optimal compromise between reaction rate, raw material cost, and processing efficiency. Temperature was identified as the sole variable governing product selectivity, with substrate-dependent optimal windows: for betulin, 〈 0 °C yields the C3-alcohol, while 〉 20 °C yields the C3-OTFA ester; for betulinic acid, 〈 –20 °C yields the C3-alcohol and 〉 10 °C yields the C3-OTFA ester, reflecting its distinct steric and electronic environment; for betulonic acid, no esterification occurs at any temperature due to the C3-ketone, making temperature control exclusively govern rearrangement efficiency.

### General procedure for TFA-mediated rearrangement

To a stirred solution of the starting material (1.0 mmol) in dichloromethane (20 mL), trifluoroacetic acid (0.2 mL, 99%, 2.6 mmol) was added at the specified temperature. The reaction progress was monitored by TLC (idione vapor). Ambient-temperature reactions typically reached completion within 4 h, while low-temperature reactions required up to 10 h to achieve full conversion, with the exact endpoint determined by TLC monitoring every 1 h. Upon completion, the mixture was washed sequentially with water and saturated aqueous NaHCO₃. The organic layer was separated, dried over anhydrous Na₂SO₄ (5 g), filtered, and concentrated under reduced pressure. The crude product was obtained in high purity (>95% by NMR) and used without further chromatographic purification.

### Temperature-dependent product selectivity

**Scheme 1:**
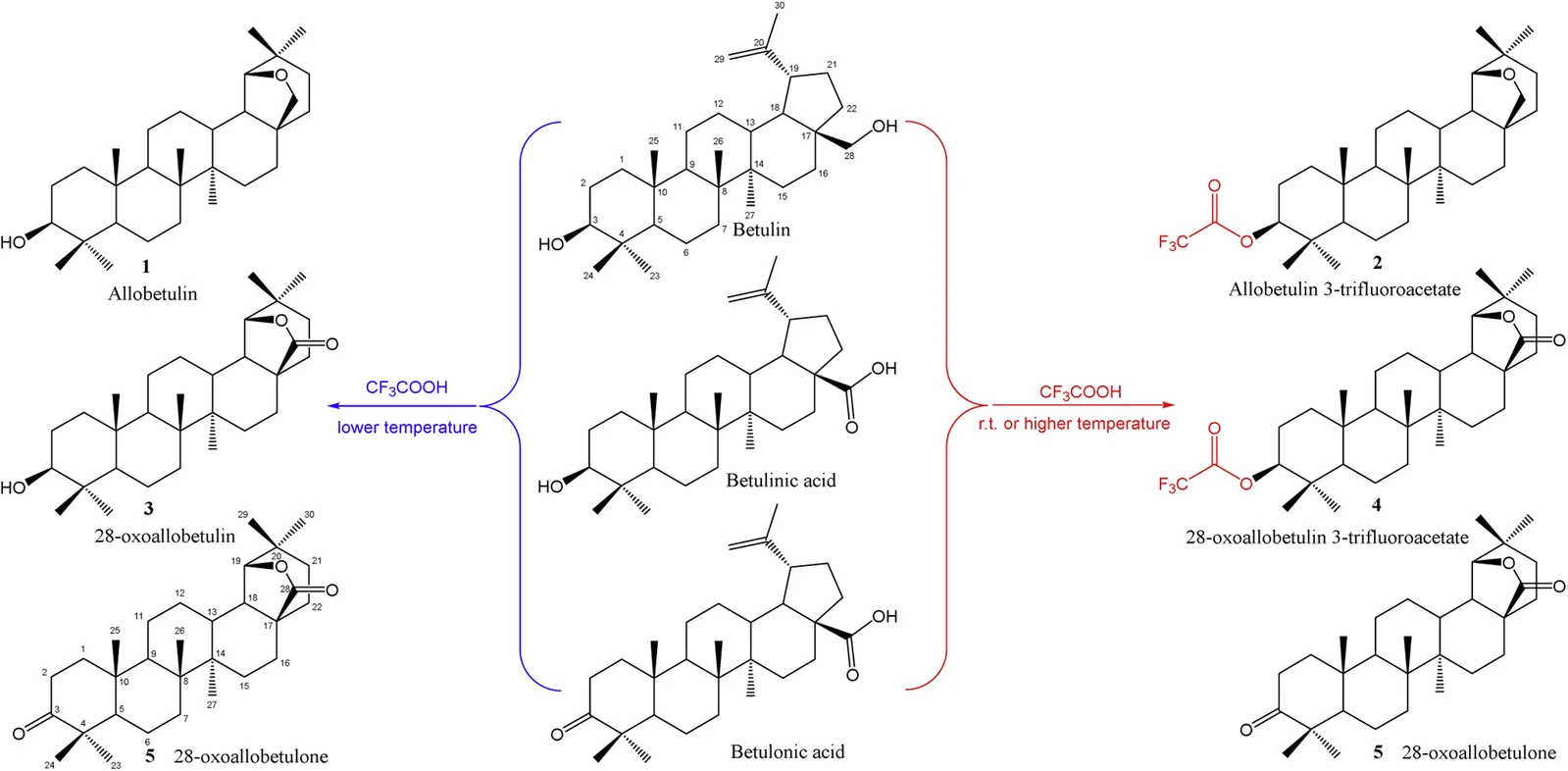
Temperature-controlled, TFA-mediated selective synthesis of allobetulin derivatives.

**Scheme 2:**
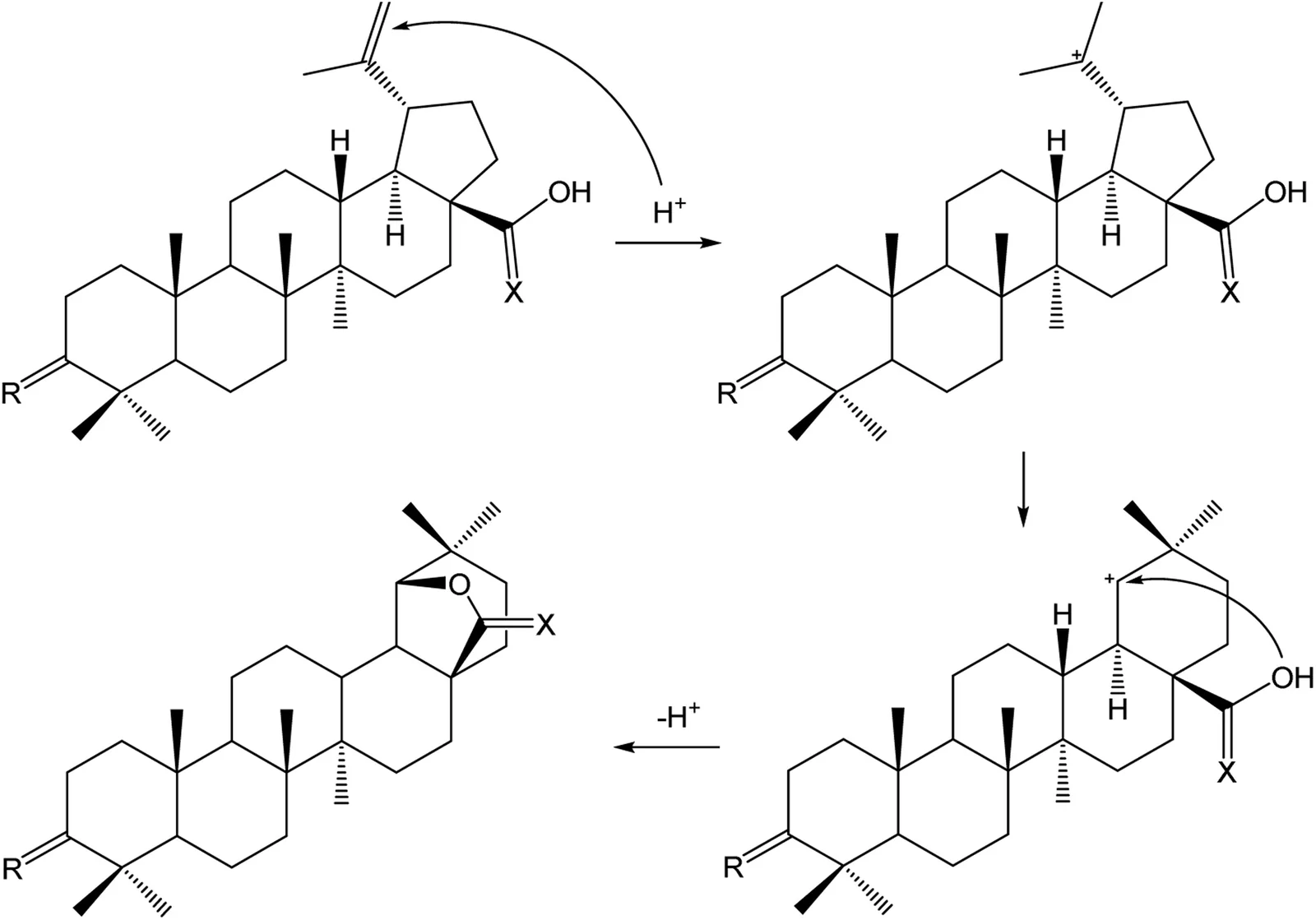
Proposed mechanism for TFA-mediated Wagner-Meerwein rearrangement.

The selectivity of the rearrangement is strictly controlled by the reaction temperature, allowing for the targeted synthesis of either free alcohols or trifluoroacetate esters (Scheme 1). As illustrated in Scheme 2, TFA first catalyzes the Wagner-Meerwein rearrangement at C-20 to furnish the allobetulin skeleton. Since the C-28 hydroxyl is consumed during the rearrangement, the product distribution is dictated solely by the fate of the C-3 hydroxyl. At low temperature, TFA lacks sufficient thermal energy to esterify the C-3 hydroxyl, thus the C3-free alcohol is obtained. At ambient temperature, thermal activation enables TFA to react with the C-3 hydroxyl, yielding the C3-O-trifluoroacetate ester. Therefore, temperature acts as a switch to control the esterification of the C-3 hydroxyl, enabling reagent-free selectivity between the C3-alcohol and C3-ester.

### Synthesis of C-3 free alcohols (Low temperature protocol)

• **Allobetulin (1):** Starting from betulin at **0 **°**C**. Yield: 93.0%. TLC (EA/PE = 1/7, R_f_ = 0.30). ^1^H NMR (CDCl_3_, 400 MHz) [8,12] *δ*: 3.77 (d, *J* = 7.8 Hz, 1H), 3.52 (s, 1H), 3.43 (d, *J* = 7.8 Hz, 1H), 3.19 (dd, *J* = 11.1, 5.1 Hz, 1H), 1.71 (dt, *J* = 13.1, 3.6 Hz, 1H), 0.97 (s, 6H), 0.92 (s, 3H), 0.91 (s, 3H), 0.84 (s, 3H), 0.79 (s, 3H), 0.76 (s, 3H), 0.69 (d, *J* = 9.4 Hz, 1H). ^13^C NMR (CDCl_3_, 100 MHz) *δ*: 87.9, 78.9, 71.2, 55.5, 51.0, 46.8, 41.4, 40.7, 40.6, 38.9, 38.9, 37.2, 36.7, 36.2, 34.1, 33.9, 32.7, 28.8, 28.0, 27.4, 26.4, 26.4, 26.2, 24.5, 21.0, 18.2, 16.5, 15.7, 15.4, 13.5.
• **28-oxoallobetulin (3):** Starting from betulinic acid at **−20 **°**C**. Yield: 90.0%. TLC (EA/PE = 1/7, R_f_ = 0.25). ^1^H NMR (CDCl_3_, 400 MHz) *δ*: 3.94 (s, 1H), 3.20 (dd, *J* = 11.3, 5.0 Hz, 1H), 1.80 (d, *J* = 11.1 Hz, 1H), 1.03 (s, 3H), 0.97 (s, 3H), 0.95 (s, 3H), 0.91 (s, 3H), 0.87 (s, 3H), 0.84 (s, 3H), 0.76 (s, 3H), 0.69 (dd, *J* = 11.2, 1.9 Hz, 1H). ^13^C NMR (CDCl_3_, 100 MHz) *δ*: 179.8, 86.0, 78.9, 55.5, 51.2, 46.7, 46.1, 40.6, 39.9, 38.9, 38.9, 37.3, 36.0, 33.7, 33.5, 32.3, 31.9, 28.7, 27.9 27.9, 27.4, 26.5, 25.5, 23.9, 20.9, 18.1, 16.5, 15.5, 15.3, 13.6. HRMS (ESI) calcd for C_30_H_49_O_3_ [M+H]^+^ 457.3682, found 457.36780.

### Synthesis of C-3 trifluoroacetate esters (ambient temperature protocol)

• **Allobetulin 3-Trifluoroacetate (2):** Starting from betulin at **23 **°**C**. Yield: 95.0%. TLC (EA/PE = 1/7, R_f_ = 0.61). ^1^H NMR (CDCl_3_, 400 MHz) *δ*: 4.68 (dd, *J* = 11.0, 5.4 Hz, 1H), 3.77 (d, *J* = 7.7, 1.2 Hz, 1H), 3.52 (s, 1H), 3.44 (d, *J* = 7.8 Hz, 1H), 0.98 (s, 3H), 0.93 (s, 3H), 0.92 (s, 3H), 0.89 (s, 9H), 0.80 (s, 3H). ^13^C NMR (CDCl_3_, 100 MHz) *δ*: 157.3 (q, *J* = 41.6 Hz), 114.6 (q, *J* = 286.1 Hz), 87.9, 86.2, 71.2, 55.4, 50.9, 46.8, 41.4, 40.7, 40.6, 38.4, 38.0, 37.1, 36.7, 36.2, 34.1, 33.7, 32.6, 28.8, 27.7, 26.4, 26.3, 26.2, 24.5, 23.2, 21.0, 18.0, 16.5, 16.2, 15.6, 13.4. HRMS (ESI) calcd for C_32_H_50_O_3_F_3_ [M+H]^+^ 539.3712, found 539.3725.
• **28-oxoallobetulin 3-Trifluoroacetate (4):** Starting from betulinic acid at **20 **°**C**. Yield: 95.0%. TLC (EA/PE = 1/7, R_f_ = 0.50). ^1^H NMR (CDCl_3_, 400 MHz) *δ*: 4.68 (dd, *J* = 10.8, 5.6 Hz, 1H), 3.94 (s, 1H), 1.81 (d, *J* = 11.1 Hz, 1H), 1.03 (s, 3H), 0.96 (s, 3H), 0.92 (s, 3H), 0.89 (s, 9H), 0.88 (s, 3H), 0.82 (dd, *J* = 11.3, 2.1 Hz, 1H). ^13^C NMR (CDCl_3_, 100 MHz) *δ*: 179.8, 157.4 (q, *J* = 41.7 Hz), 114.6 (q, *J* = 286.2 Hz), 86.2, 86.0, 55.4, 51.1, 46.7, 46.1, 40.6, 39.9, 38.4, 38.1, 37.1, 36.0, 33.6, 32.3, 31.9, 28.7, 27.9, 27.7, 26.4, 25.5, 23.9, 23.2, 20.9, 17.9, 16.6, 16.2, 15.5, 13.6. HRMS (ESI) calcd for C_32_H_48_O_4_F_3_ [M+H]^+^ 553.3505, found 553.34939.
• **28-oxoallobetulone (5):** Starting from betulonic acid at **23 **°**C**. Yield: 95.0%. TLC (EA/PE = 1/7, R_f_ = 0.33). ^1^H NMR (CDCl_3_, 400 MHz) *δ*: 3.95 (s, 1H), 2.37–2.57 (m, 2H), 1.94 (ddd, *J* = 12.6, 7.5, 4.7 Hz, 1H), 1.83 (d, *J* = 11.1 Hz, 1H), 1.07 (s, 3H), 1.03 (s, 3H), 1.03 (s, 3H), 0.96 (s, 3H), 0.95 (s, 3H), 0.94 (s, 3H), 0.89 (s, 3H). ^13^C NMR (CDCl_3_, 100 MHz) *δ*: 217.9, 179.7, 85.8, 54.8, 50.4, 47.2, 46.5, 46.0, 40.3, 39.8, 39.7, 36.8, 36.0, 33.9, 33.4, 32.8, 32.2, 31.8, 28.6, 27.7, 26.6, 26.4, 25.4, 23.8, 21.3, 20.8, 19.4, 16.3, 15.2, 13.5. HRMS (ESI) calcd for C_30_H_47_O_3_ [M+H]^+^ 455.3525, found 455.35247.

### Product characterization

Structural identity and purity of all isolated compounds were confirmed by ¹H/¹³C NMR spectroscopy.

Key diagnostic signals for product differentiation:

C3 free alcohols: the C3 proton resonates at δ ∼3.19 ppm as a doublet of doublets (*J* = 11.0, 5.0 Hz).

C3 trifluoroacetate esters: the C3 proton resonates at δ ∼4.68 ppm as a doublet of doublets.

Wagner-Meerwein rearrangement: The disappearance of the two terminal alkene proton signals at δ 4.5–4.7.

## Method validation

The robustness of the temperature-controlled protocol was validated across three structurally distinct lupane-type triterpenoid substrates, as summarized in Table 1. For betulin, the low-temperature protocol (<10 °C) afforded allobetulin (1) in 93% isolated yield, while the ambient-temperature protocol (23 °C) yielded allobetulin 3-trifluoroacetate (2) in 95% isolated yield. For betulinic acid, the low-temperature protocol (−20 °C) gave 28-oxoallobetulin (3) in 90% isolated yield, and the ambient-temperature protocol (20 °C) produced 28-oxoallobetulin 3-trifluoroacetate (4) in 95% isolated yield. For betulonic acid, the ambient-temperature protocol (23 °C) delivered 28-oxoallobetulone (5) in 95% isolated yield.

**Table 1:** Substrate Scope and Isolated Yields of the TFA-Mediated Wagner-Meerwein Rearrangement.

**Entry**	**Starting Material**	**Temp ( **°C**)**	**Product (Compound No.)**	**Yield (%)**
1	Betulin	0	Allobetulin (**1**)	93
2	Betulin	23	Allobetulin 3-trifluoroacetate (**2**)	95
3	Betulinic acid	−20	28-oxoallobetulin (**3**)	90
4	Betulinic acid	20	28-oxoallobetulin 3-trifluoroacetate (**4**)	95
5	Betulonic acid	23	28-oxoallobetulone (**5**)	95

*Note:* Betulinic acid (entry 4) and betulonic acid (entry 5) are not interchangeable; the latter features a C-3 ketone in place of the C-3 hydroxyl.

All isolated products exhibited >95% purity by ¹H NMR analysis, eliminating the need for chromatographic purification that typically causes 10–30% product loss in traditional methods [13]. The crude products were sufficiently pure for most downstream derivatization reactions, and recrystallization from methanol/water provided material suitable for spectroscopic characterization.

## Limitations

• Strict temperature control is critical for selective synthesis of C3 free alcohol allobetulin derivatives. For betulinic acid, the reaction must be maintained at <−20 °C to avoid formation of the C3 trifluoroacetate ester; deviations to −10 °C result in a mixture with the ester byproduct. For betulin, the threshold for pure C3-alcohol is <0 °C.
• The protocol is currently validated for batch sizes up to 5 mmol; scale-up to > 5 mmol may require modified heat dissipation to maintain precise temperature control.
• Currently limited to lupane-type triterpenoids from betulin/betulinic acid/betulonic acid; extension to other scaffolds remains to be tested

## Ethics statements

None.

## Related research article

Y. Wang, X. Huang, X. Zhang, J. Wang, K. Li, G. Liu, K. Lu, X. Zhang, C. Xie, T. Zheng, T., Y.-Y. Cheng, Q. Wang, Synthesis and biological evaluation of novel allobetulin/allobetulin-nucleoside conjugates as antitumor agents, Molecules 27(2022)4738. https://doi.org/10.3390/molecules27154738

## CRediT authorship contribution statement

**Yanli Wang:** Investigation, Methodology, Formal analysis, Writing – original draft, Visualization. **Panpan Sun:** Investigation, Validation. **Li Wang:** Investigation. **Xiwei Zhao:** Investigation. **Gang Huang:** Investigation. **Xiaofei Hao:** Investigation. **Peng Liu:** Project administration, Supervision, Writing – review & editing. **Qiang Wang:** Conceptualization, Methodology, Resources, Funding acquisition, Writing – review & editing.

## Declaration of competing interest

The authors declare the following competing interest: A related Chinese patent application (No. 202,610,350,664.3) has been filed by the authors, covering the synthetic protocol described in this work.

## Data Availability

Data will be made available on request.

## Appendix Supplementary materials

Image, application 1
