# Supplementary material for: Temperature-controlled, chromatography-free synthesis of allobetulin derivatives via trifluoroacetic acid-mediated Wagner-Meerwein rearrangement
Source: MethodsX. 2026 Aug 25;17:104115. doi: 10.1016/j.mex.2026.104115 (PMC13545346; doi:10.1016/j.mex.2026.104115)
Supplement: Supplementary file 1 [file mmc1.docx]

**Temperature-Controlled, Chromatography-Free Synthesis of Allobetulin Derivatives via Trifluoroacetic Acid-Mediated Wagner-Meerwein Rearrangement**

Yanli Wang^a,b^, Panpan Sun^b^, Li Wang^c^, Xiwei Zhao^b^, Gang Huang^d^, Xiaofei Hao^d^, Peng Liu^a,d∗^, Qiang Wang^c,d,∗^

*^a^ School of Chemical Engineering, Zhengzhou University, Zhengzhou, 450001, China*

*^b^ Institute of Reproductive Health, Henan Academy of Innovations in Medical Science, Zhengzhou, 453000, China*

*^c^ School of Medicine, Huanghe Science and Technology College, Zhengzhou, 450063, China*

*^d^ Institute of Chemistry, Henan Academy of Sciences, Zhengzhou, 450002, China*

*^e^ Quality Inspection and Analysis Testing Research Center, Henan Academy of Sciences, Zhengzhou, 450002, China*

#### Corresponding author: liupeng615128@hnas.ac.cn; qiangwang@hnas.ac.cn

16 figures

Fig. SI-1. ^1^H NMR spectrum of compound 1

Fig. SI-2. ^13^C NMR spectrum of compound 1

Fig. SI-3. ^1^H NMR spectrum of compound 2

Fig. SI-4. ^13^C NMR spectrum of compound 2

Fig. SI-5. HRMS spectrum of compound 2

Fig. SI-6. ^1^H NMR spectrum of compound 3

Fig. SI-7. ^13^C NMR spectrum of compound 3

Fig. SI-8. HRMS spectrum of compound 3

Fig. SI-9. ^1^H NMR spectrum of compound 4

Fig. SI-10. ^13^C NMR spectrum of compound 4

Fig. SI-11. HRMS spectrum of compound 4

Fig. SI-12. ^1^H NMR spectrum of compound 5

Fig. SI-13. ^13^C NMR spectrum of compound 5

Fig. SI-14. HRMS spectrum of compound 5

Fig. SI-15. ^1^H NMR spectrum of the reaction mixture of betulin with TFA at 10℃ after 24h

Fig. SI-16. ^1^H NMR spectrum of the reaction mixture of betulinic acid with TFA at -10℃ after 24h
